# Refraction development in anisometropic amblyopia with patching therapy

**DOI:** 10.3389/fmed.2022.959085

**Published:** 2022-10-18

**Authors:** Yao Chen, Jingjing Zuo, Yue Xiong, Xi Yu, Lili Wei, Yifan Luo, Jinhua Bao, Hao Chen, Jiawei Zhou

**Affiliations:** School of Ophthalmology and Optometry, Affiliated Eye Hospital, State Key Laboratory of Ophthalmology, Optometry and Vision Science, Wenzhou Medical University, Wenzhou, China

**Keywords:** anisometropia, amblyopia, anisometropic amblyopia, myopic shift, emmetropization, patching therapy

## Abstract

**Purpose:**

To investigate the development of refraction in anisometropic amblyopia who had been with patching therapy.

**Methods:**

We retrospectively reviewed 37,528 medical records of the amblyopes who had been treated with patching therapy between July 2003 and January 2020 at the School of Optometry and Ophthalmology and Eye Hospital of Wenzhou Medical University. We included unilateral anisometropic amblyopia with a follow-up length of not < 2 years. In total, 371 cases were enrolled and followed up for a mean of 4.76 ± 2.11 years. The subjects were then divided into different groups and periods according to their initial spherical equivalent (SE) refractive error and best-corrected visual acuity (BCVA) of the amblyopic eye. Linear mixed-effects models were fitted to calculate the annual change of SE.

**Results:**

The annual changes in SE were −0.32 (−0.35 to −0.30) and −0.16 (−0.19 to −0.14) D/yr for the amblyopic eye and the fellow eye, respectively. The annual changes in SE of amblyopic eyes during the treatment period and the successfully treated period were −0.36 (−0.43 to −0.29; 95% CI) and −0.27 (−0.32 to −0.23; 95% CI) D/yr, respectively; the annual SE changes of the fellow eye during the treatment period and the successfully-treated period were −0.07 (−0.14 to −0.01; 95% CI) and −0.18 (−0.22 to −0.14; 95% CI) D/yr, respectively.

**Conclusion:**

The amblyopic eye experienced a significantly greater degree of refractive error changes than the fellow eye and underwent a continuous refractive error reduction before and after 7 years old. After the patching therapy was terminated, emmetropization in the amblyopic eye remained synchronized, whereas the refractive error change was increased in the fellow eye.

## Introduction

Approximately 90% of newborn infants are born with hyperopia [generally in the range of +2 to +3.5 diopters (D)] (1, 2), which decreases significantly during the first 2 years of life (3). This process of mitigating hyperopia is known as emmetropization, which results in the state of normal refractive condition of both eyes during the developmental period (4). Emmetropization is an active process. It is regulated by visual input and affects the visual system of the eyes' refractive condition (5). If there is abnormal visual development, the process of emmetropization can also be abnormal. It can be associated with clinically significant refractive error (6). An asynchronous emmetropization can induce a significant and persistent anisometropia, which is known to be a high-risk factor for amblyopia (7) and strabismus (8, 9).

Amblyopia is a neurodevelopmental disorder with no ocular pathology. It originates from abnormal visual experiences during childhood (10). Amblyopia affects 1–5% of the population (11, 12). Eighty to 90% of amblyopes have a significant refractive error (myopia, hyperopia, or astigmatism) (13, 14), 37% of which can be attributed to anisometropic amblyopia (15). Anisometropic amblyopia can be found in individuals who have experienced defocus retinal image (16) and active suppression (17) from their refractive errors. There has been evidence that severe anisometropia can increase the likelihood of anisometropic amblyopia occurrence (18). Also, stereoacuity deficits in amblyopia are highly associated with the magnitude of anisometropia (19). In sum, these studies suggest that the refraction development (optical system) is highly pertinent to amblyopia.

Patching therapy, which involves occluding the fellow eye to enforce the amblyopic eye to work, has been standard treatment for amblyopia in the clinic (20). Visual acuity has been used as the primary outcome measure to determine whether the patient experiences an adequate visual recovery after patching (21). However, whether the end-state of refraction in both amblyopic and fellow eyes could be affected by patching therapy is unknown. This issue is important not only because amblyopes exhibit abnormal refractive errors but also because the patching therapy is normally conducted during the critical period when refraction development also takes place. In particular, the treatment period for patching therapy for amblyopia is within 8 years after birth (22). This period coincides with emmetropization (23, 24). To illustrate, studies report that children aged 6–8 years old experience a significant refractive error change of ~-0.3 D/yr, whereas children aged 9–13 years old merely show a refractive error change of ~-0.1 D/yr (24). Moreover, animal studies show that guinea pigs develop deprivation myopia if they have worn a diffuser on one eye during the critical period of visual development (25). This indicates that deprivation of an eye can interfere with the emmetropization of guinea pigs. For these reasons, it seems highly probable that patching therapy can interfere with the refractive development during the critical period of visual development.

However, the current understanding of refraction development in amblyopia is limited due to contradictory findings from previous studies. For instance, Cecil et al. conducted a retrospective study to compare the refractive error changes of 55 strabismic individuals with unilateral amblyopia (aged from 6 months to 9 years old). They followed patients from 5 to 29 years. The study shows that the fellow, rather than the amblyopic, eye is more likely to develop myopia (26). Similar findings have been reported in subsequent studies (27, 28). However, Park et al. analyzed the first 12 years of follow-up data and reported that the amblyopic eye with accommodative esotropia can experience a significantly greater decrease in spherical equivalent (SE) over time than the non-amblyopic eye (29). Also, Shinh et al. followed patients with anisometropic amblyopia (≥ 3D) and made an observation that the myopization in those with hyperopic amblyopia is synchronous in both eyes (30). In sum, these studies show that the amblyopic and fellow eyes have different patterns of myopia development.

Unfortunately, the findings from the previous studies do not enable us to parse the influence of patching therapy on the development of refractive error from other external factors due to designs, inadequate sample size (*n* = 30~120) and the inclusion of individuals with mixed strabismus. Another issue with the designs is that longitudinal data in individuals with anisometropic amblyopia beyond 7 years are lacking; it is considered a turning point of the refractive development (31). In this study, we review the clinical data of 371 anisometropic amblyopes and explore the patterns of refraction development until 15 years to answer these research questions: (1) What is the pattern of refractive development in anisometropic amblyopia during the patching therapy? (2) Are there any differences in the refractive development after the termination of the patching therapy from a proper visual recovery? (3) Is there any difference in the refractive development before and after 7 years old? Since both the initial hyperopia during development (32, 33) and clear vision (34) can affect emmetropization, we hypothesized that the amblyopic eye could experience a more myopic shift than the fellow eye and that the increase of myopic shift from the development of amblyopia could be successfully treated.

## Methods

The study adhered to the tenets of the Declaration of Helsinki and was approved by the School of Optometry and Ophthalmology and Eye Hospital of Wenzhou Medical University. We retrospectively reviewed the medical records of the treatment process of 37,528 cases between 2003 and 2020. We recruited children who had (1) unilateral anisometropic amblyopia detected and were followed up not < 2 years, (2) the spherical equivalent refraction (SE; the sum of the spherical and the half of the cylinder) in the amblyopic eye ≥ 4 D, (3) the cylinder of both eyes ≤ 3 D. Exclusion criteria included glaucoma, cataract, ptosis, bilateral amblyopia, macular diseases, systemic diseases, and a history of ocular surgery. Unilateral anisometropic amblyopia were defined as the following criteria: the interocular difference of best-corrected visual acuity (BCVA) being more than 2-lines, BCVA in the amblyopic eye > 0.1 logMAR (35), and a significant anisometropia of ≥ 1 D SE between the two eyes. We categorized their visual treatment as successful if they ended up having a BCVA ≤ 0.1 logMAR and no regression during the following visits, a difference between two eyes < 2 lines, and displayed a sustained visual improvement for at least 3 months (36).

All subjects were examined by ophthalmologists and optometrists. Anterior segment was examined by slit lamp; BCVA was tested by tumbling “E” chart; cycloplegic refraction, prism alternate cover testing, and Krimsky was used for ocular alignment for distance and near; anterior segment examination, fundoscopy evaluation, and type of amblyopia were carried out at the initial visit. BCVA, refraction, and ocular alignment were examined at each visit. Most subjects received 1% atropine during their first visit upon diagnosis and 1% tropicamide or 1% cyclopentolate during their following visits. It has been shown that the difference between cycloplegic refractions acquired by 1% tropicamide or 1% cyclopentolate is not significant (37). To reduce the confounding effects of different cycloplegic agents, we only included cyclopentolate refraction that was obtained using 1% tropicamide or 1% cyclopentolate during patients' following visits in our data analysis (38). The initial SE refers to the first non-atropine cycloplegic refraction. All amblyopes were prescribed with wearing spectacles all day and patching (2–6 h/day) upon their diagnosis of amblyopia.

### Statistical analysis

All statistical tests were conducted using RStudio (Copyright2009–2019 RStudio, Inc.). In this paper, continuous data are presented as mean ± SD. These were analyzed using the Mann-Whitney U tests due to their skewed distributions. Categorical data are presented as the number of cases (%) and a chi-square test was used to compare the differences between the two groups. Regarding the chi-square test, the effect size is reported as Ψ. The annual rate of changes and 95% confidence interval (CI) in refraction were estimated from mixed-effect models, where the age change was modeled as a continuous variable to get the slope estimate. For fixed effects, we included the initial refractive error in the model. As random effects, we included intercepts for subjects and times, as well as by-subject and by-time random slopes for the change of age. Subgroup analyses of moderate hyperopic amblyopia and high hyperopic amblyopia were also performed. The *Z*-test was utilized to compare the difference between subgroups. The effect sizes for Mann-Whitney U tests and Z tests are reported as *r* (39). All statistical tests were two-sided, and the *p*-value ≤ 0.05 was deemed as statistically significant.

## Results

### Clinical demographics

Three hundred and seventy one subjects (225 males, 146 females) who met the inclusion criteria were included in the data analysis. Their mean age was 6.50 ± 2.81 years old (range from 1 to 15 years old). They were followed up until 15 years old, with a mean follow-up period of 4.76 ± 2.11 years. At the initial visit, the SE was 5.57 ± 1.21 D and 0.88 ± 1.11 D for amblyopic eyes and fellow eyes, respectively. The BCVA was 0.61 ± 0.32 logMAR and 0.02 ± 0.07 logMAR for amblyopic eyes and fellow eyes, respectively. The demographic characteristics of hyperopic amblyopia are shown in Table 1.

**Table 1:** Demographic characteristics of the patients.

	**All (*n* = 371)**	**Grouped by refractive error of the amblyopic eye at the first visit**			
		**Moderate hyperopia (*n* = 225)**	**High hyperopia (*n* = 146)**	***P-*value**	**Effect size (*r*)**
Age at the first visit, years	6.50 ± 2.81	6.60 ± 2.90	6.33 ± 2.67	0.422	0.042
Male, (%)	225 (60.65)	141 (62.67)	84 (57.53)	0.323	0.051*
Follow up duration, years	4.76 ± 2.11	4.67 ± 2.03	4.91 ± 2.22	0.368	0.047
SE of AE (D) at the first visit	5.57 ± 1.21	4.77 ± 0.65	6.80 ± 0.77	<0.001	0.846
BCVA of AE at the first visit	0.61 ± 0.32	0.54 ± 0.28	0.72 ± 0.35	<0.001	0.254
SE of FE (D) at the first visit	0.88 ± 1.11	0.68 ± 0.90	1.19 ± 1.31	0.002	0.163
BCVA of FE at the first visit	0.02 ± 0.07	0.02 ± 0.07	0.03 ± 0.08	0.582	0.029
Anisometropia (D) at the first visit	4.69 ± 1.40	4.09 ± 1.07	5.61 ± 1.35	<0.001	0.531
SE of AE (D) at the last visit	4.05 ± 1.61	3.36 ± 1.21	5.12 ± 1.58	<0.001	0.546
SE of FE (D) at the last visit	0.15 ± 1.35	−0.07 ± 1.26	0.48 ± 1.41	<0.001	0.198
Anisometropia (D) at the last visit	3.90 ± 1.59	3.43 ± 1.31	4.64 ± 1.71	<0.001	0.372

SE, spherical equivalent refraction, which was calculated by the sum of the spherical and half of the cylinder.

BCVA, best-corrected visual acuity converted to logarithm of the minimal angle of resolution (logMAR).

AE, amblyopic eye; FE, fellow eye.

Continuous data were presented as mean ± SD, compared by the Mann-Whitney U-tests.

Categorical data were presented as the number of cases (%) and the chi-square tests were utilized to compare the differences between the two groups. The asterisk (^*^) represents the effect of chi-square test (Ψ).

P-values represent the differences between moderate hyperopia and high hyperopia.

### Refractive error changes in amblyopic eyes and fellow eyes

**Figure 1:**
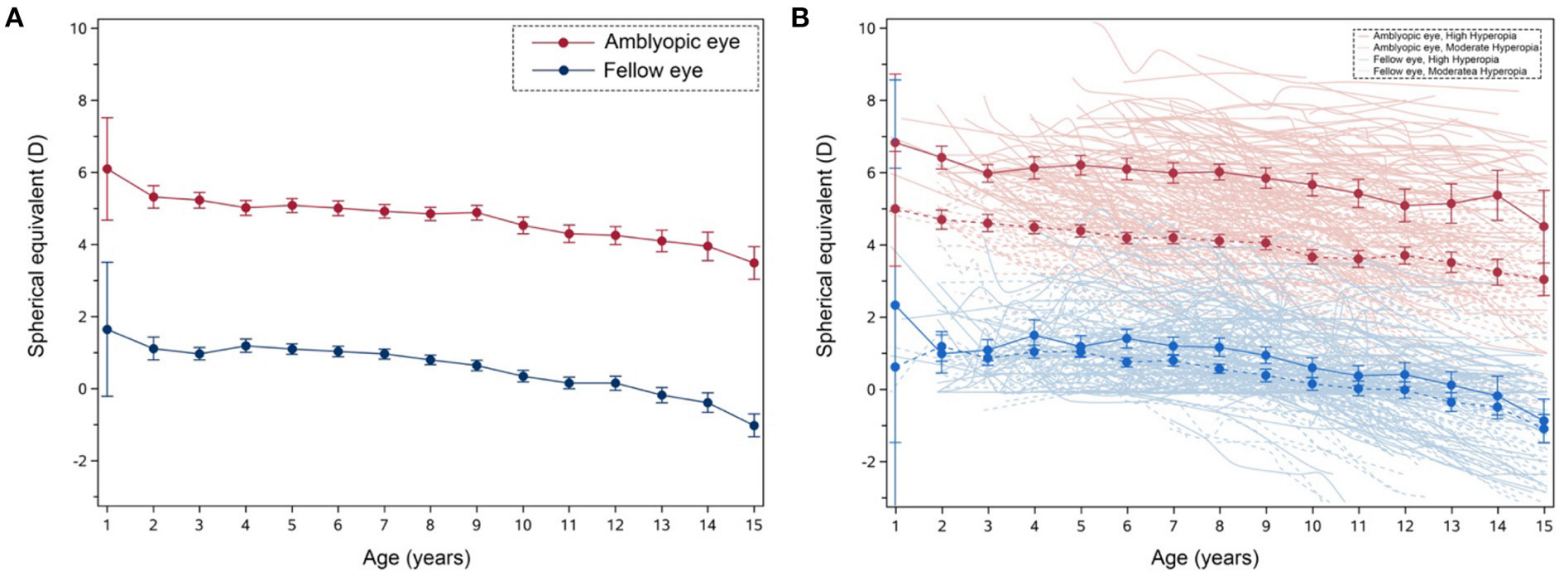
The tendency of changes in the mean spherical equivalent (SE) refractive error in 371 cases of anisometropic amblyopia over time. **(A)** The red solid line represents the amblyopic eye, and the blue solid line represents the fellow eye. Error bars represent standard errors. **(B)** The dark red line represents the mean refraction of the amblyopic eye in the high hyperopic group and the dark blue line represents the mean refraction of the fellow eye in the high hyperopic group. The dark dash red line represents the mean refraction of the amblyopic eye in the moderate hyperopic group and the dark dash blue line represents the mean refraction of the fellow eye in the moderate hyperopic group. Error bars represent standard errors. The light red line represents the amblyopic eye in the high hyperopic group and the light blue line represents the fellow eye in the high hyperopic group. The light dash red line represents the amblyopic eye in the moderate hyperopic group and the light dash blue line represents the fellow eye in the moderate hyperopic group.

We found a general decline in SE during the follow-up period in both eyes (Figure 1A). The rate of changes in SE (95%CI) was −0.32 (−0.35 to −0.30) and −0.16 (−0.19 to −0.14) D/yr for the amblyopic and fellow eyes, respectively. There was a significant difference between the amblyopic eye and the fellow eye in terms of their annual changes [*p* < 0.01, *Z*-test (*r* = 0.353)].

Previous studies indicate that a greater refractive error change is associated with a higher initial level of hyperopia (32, 33). However, we found the correlation between the initial SE and annual change was not significant, *r* = −0.07, *p* = 0.17 (Spearman correlation analysis). According to the categories in Hu et al. (38), we defined SE ≥ +4 D and < +6 D as moderate group, ≥ +6 D as high group. Figure 1B shows the change in SE as a function of patients' age and estimated linear regression lines of the moderate hyperopic group and the high hyperopic group, respectively. We found that in the moderate hyperopic group, the mean rate of changes in SE was −0.31 (−0.34 to −0.27) and −0.16 (−0.19 to −0.13) D/yr for amblyopic eyes and fellow eyes, respectively. In the high hyperopic group, we found that the mean rate of changes in SE was −0.35 (−0.40 to −0.30) and −0.16 (−0.20 to −0.12) D/yr for amblyopic eyes and fellow eyes, respectively. There was neither a statistical difference between the refractive error change rate of the high hyperopic group and the moderate hyperopic group [*p* = 0.154, *Z*-test (*r* = 0.074)], nor between the fellow eyes of the two groups [*p* = 0.951, *Z*-test (*r* = 0.003)].

**Figure 2:**
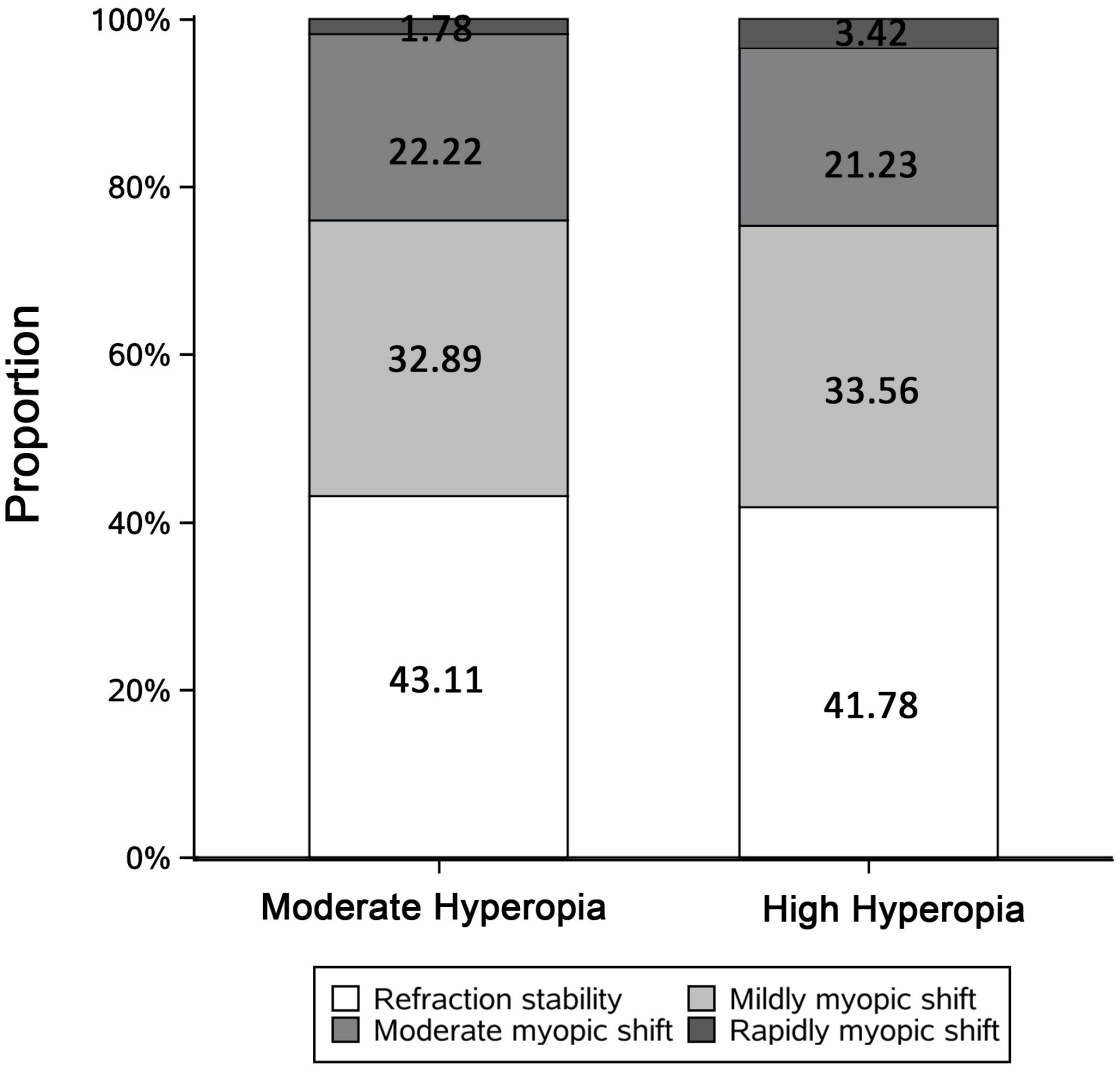
Bar graph showing distributions of the patterns of refraction development by initial SE. Refraction stability, rate of change in SE < ±0.25 D/year; Mildly myopic shift, rate of change in SE > −0.50 and ≤ −0.25 D/year; Moderately myopic shift, rate of change in SE > −1.00 and ≤ −0.50 D/year; Rapidly myopic shift, rate of change in SE ≤ −1.00 D/year. The numbers represent the proportions of each part.

To better illustrate the patterns of the refractive error change in the moderate and high hyperopic group, we decided to define the patterns of refraction development based on the annual change of SE, including refraction stability (annual change of SE < ±0.25 D/year), mild myopic shift (annual change of SE > −0.50 D and ≤ −0.25 D/year), moderate myopic shift (annual change of SE > −1.00 D and ≤ −0.50 D/year), and rapid myopic shift (annual change of SE, < −1.00 D/year) (38). Figure 2 shows a similar proportion pattern of myopillization in both groups.

### Refractive error changes before and after 7 years old

To illustrate the effect of age on refractive error changes, we grouped these patients based on the initial age and compared the mean SE changes between amblyopic and fellow eyes. We found a general decline tendency in SE before and after 7 years of age. The difference in SE changes between < 7 years and ≥ 7 years in the amblyopic eye [*p* = 0.502, *Z*-test (*r* = 0.035)] was not significant, but it was significant in the fellow eye [*p* < 0.001, *Z*-test (*r* = 0.203)]. In the moderate hyperopia group, the annual changes in SE in the amblyopic eye < 7 years old and ≥ 7 years old were −0.28 (−0.32 to −0.24; 95% CI) and −0.34 (−0.39 to −0.29; 95% CI), respectively. The annual SE changes of the fellow eye were −0.11 (−0.15 to −0.07; 95% CI) and −0.22 (−0.26 to −0.17; 95% CI), respectively. In the high hyperopia group, the annual change of SE of amblyopic eyes aged < 7 years old and ≥ 7 years old was −0.36 (−0.44 to −0.29; 95% CI) and −0.33 (−0.40 to −0.26; 95% CI), respectively. The annual SE changes of the fellow eye were −0.13 (−0.18 to −0.08; 95% CI) and −0.20 (−0.27 to −0.14; 95% CI), respectively.

### Refractive error changes in amblyopic period and successfully treated period

**Figure 3:**
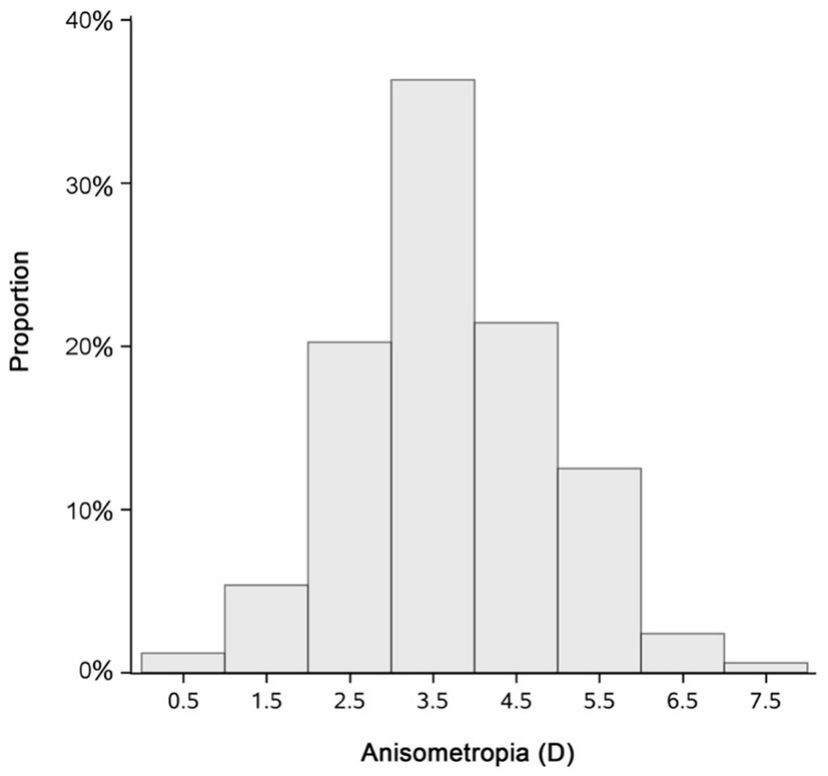
Bar graph demonstrating the anisometropia of the 168 cases when vision deficit was resolved.

**Figure 4:**
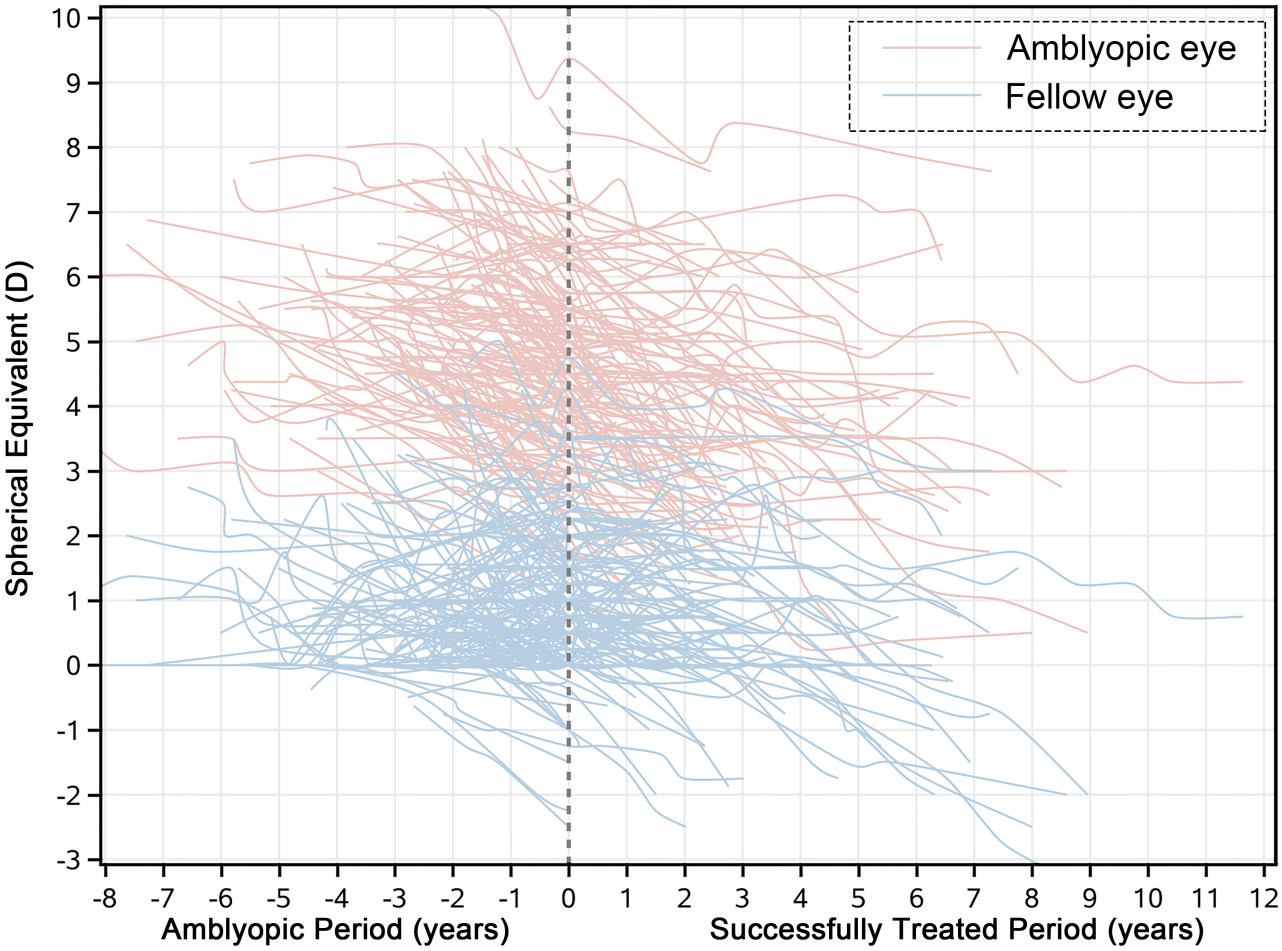
The illustration of refractive development patterns for the amblyopic period and the amblyopia resolved period. The dashed line represents the timepoint when the amblyopes were treated successfully. The light red line represents the original SE of the amblyopic eye and the light blue line represents the original SE of the fellow eye.

One hundred and sixty eight of the 371 cases that we had screened were successfully treated at the end date of the screening. At this timepoint, normal visual acuity was obtained; their mean age was 8.04 ± 3.07 years old; the mean SE was 4.57 ± 1.40 D for the amblyopic eye, and the mean anisometropia was 3.64 ± 1.22 D. Figure 3 shows the distribution of the anisometropia in the 168 cases when amblyopia was successfully treated. Next, we compared the mean SE changes during the patching therapy with that during the successfully treated period. We found that the mean rate of changes in SE was −0.36 (−0.43 to −0.29) and –0.27 (−0.32 to −0.23) D/yr for amblyopic eyes and successfully treated eyes, respectively; the mean rate of changes in SE was −0.07 (−0.14 to −0.01) and −0.18 (−0.22 to −0.14) D/yr for fellow eyes in amblyopic period and amblyopic resolved period, respectively. There was no significant difference in mean rates of changes in SE between the two periods in the amblyopic eye [*p* = 0.062, *Z*-test (*r* = 0.097)]. However, the difference in mean rates of changes in SE between the two periods in the fellow eye was significant [*p* = 0.011, *Z*-test (*r* = 0.132)]. To better illustrate the patterns of refractive development in the two periods, we plotted SE changes over time for each subject (Figure 4).

## Discussion

### Refractive error changes in amblyopic eyes and fellow eyes

In this study, we found that both eyes experienced changes in refractive error over time. This finding is in line with previous studies (26–28, 38). Our findings reveal that the amblyopic eye undergoes a greater refractive error change than the fellow eye (Figure 1). However, Wang et al. (28) observed a slower myopization in the amblyopic eye than in the fellow eye (*n* = 42). The discrepancy could be due to the difference in anisometropia (4.69 D in the current study compared to 0.59 D in Wang et al.) and types of amblyopia within the recruited patients, who were mostly accommodative esotropes or bilateral amblyopes in the previous study. In our study, however, all the participants were anisometropic individuals with unilateral amblyopia.

Some other studies report a similar refractive error change between the amblyopic eye and the fellow eye. Atilla et al. (40) and Shih et al. (30) reported that the amblyopic eye and the fellow eye experience a slight decrease in synchronization with a similar refractive error change of −0.2 D/yr in both eyes. On the other hand, our findings report that the mean rate of changes in SE was −0.32 and −0.16 D/yr for the amblyopic eye and the fellow eye, respectively. The higher the initial level of hyperopia, the greater the refractive error change. This trend has been reported in previous studies (32, 33). We speculate that optical defocus contributes to the trend. Due to the higher initial hyperopia of the amblyopic eye, the hypermetropic defocus can be greater in the more hyperopic eye than in the less hyperopic eye, contributing to a greater refractive error change in hyperopic eyes.

### Refractive error changes in moderate hyperopic amblyopia and high hyperopic amblyopia

Previous studies report that the refractive error change varies along with the degree of hyperopia. For instance, Hu et al. reported the mean change of SE was 0.38 and 0.45 D/yr for the moderate hyperopic group (4–6 D) and the high hyperopic group (≥ 6 D), respectively (38). We also divided our subjects into two groups based on the initial SE of the amblyopic eye. However, we did not find a significant difference between the two groups. It should be noted that most subjects were first diagnosed with atropine cycloplegic refraction and were prescribed with 1% tropicamide or 1% cyclopentolate during their following visits. Since these two methods of cycloplegia led to different refractions, we used results of 1% tropicamide and 1% cyclopentolate in the subsequent analysis to make an unbiased estimation of the refractive error changes over time. Therefore, we did not group our subjects based on their atropine cycloplegic refraction from their initial visit but on their first non-atropine cycloplegic refraction after a period of treatment. This time difference was about 3 months. Such a time difference might lead to a certain bias in the grouping. For instance, for individuals with slightly high hyperopia at the initial diagnosis, the amount of hyperopia in the amblyopic eye could decrease after 3 months of treatment. Hence, they might be classified into a lower hyperopic group based on the refractive error measured after 3 months of treatment. According to Figure 1, the annual hyperopia reduction was 0.32 D/yr. This means that only those who had a refractive error between 5.9 and 6 D could be so in this case; this is not so in the case of our participants. One possibility for the difference between our findings and that of the previous literature (29, 38) could be the difference in the nature of the eye disease itself. For instance, patients reported in Hu et al. had moderate to high hyperopia in both eyes without amblyopia; patients in our study were mostly had monocular hyperopia with amblyopia (38).

### Refractive error changes before and after 7 years old

Previous studies support the notion that the critical turning point for refractive development is around 7 years of age (31). In addition, hyperopic refraction has been found to increase or remain constant from 3 to 7 years old and decrease subsequently. However, we found a decrease in refraction before and after age 7 years both in moderate and high hyperopic groups. The refractive error change in amblyopes was −0.31 and −0.16 D/yr in both eyes before 7 years old, followed by a myoplization of −0.37 and −0.18 D/yr between 7 and 15 years old in both eyes. The major reason for such a difference could be due to the inter-individual differences in subjects between our study and the previous ones. Rather than recruiting individuals with accommodative esotropia as in previous studies, we only included individuals with unilateral anisometropic amblyopia who had undergone patching therapy, which might have induced a larger and earlier change in refractive error. Our results demonstrate that amblyopic eye can undergo a similar degree of refractive error change before and after 7 years old.

### Refractive error changes in amblyopic period and successfully treated period

In our study, 168 subjects achieved a normal visual acuity after treatment. Several studies have suggested that early visual experience can critically affect the refractive development. Clear visual information through both eyes is essential emmetropization to proceed normally (34). However, our findings indicate that the amblyopic eye did not show an increased refractive error change even after blur had been entirely removed (Figure 4). The mean rate of changes in SE was −0.36 (−0.43 to −0.29) and −0.27 (−0.32 to −0.23) D/yr for the amblyopic eye and successfully treated eye. The finding could be explained by two reasons. First, as ocular component growth declines with age, due to structural limitations, a slight potential for emmetropization could remain. Second, the literature suggests that the successfully treated amblyopes can still have deficits in contrast sensitivity, stereoacuity (41) and binocular balance even if the subjects reach normal visual acuity (42). These studies collectively indicate that the pathway connection between the retina and the visual cortex (dorsal and ventral stream), along with visual feedback mechanism of retinal images, is not fully established. For the fellow eye, the myopic shift is very small during patching, which is contrary to what animal studies have shown. A study reports that Guinea pigs can develop significant myopia when their eye is viewed with a diffuser (25). This is in contrast to what human studies show, perhaps due to the difference in how patching was conducted. For instance, in the animal study, guinea pigs wore diffusers throughout the entire day, whereas the amblyopes in the other study were patched for only several hours a day. In addition, the monocular fellow eye viewing has revealed a deficit in the ocular motor function, fixation stability and motion perception (43), which cannot be regarded as totally normal (44). All these factors might play a role in giving arise to the difference between guinea pigs and amblyopes. In the present study, the fellow eye experienced a myopic shift of −0.07 D/yr during the patching period. Two recent studies in China (23, 24) show that non-amblyopic children experience a refractive error change of ~-0.2 D/yr, which might indicate that the non-patched eye can show a greater refractive error change than the patched fellow eye. In short, the refractive error change between the amblyopic period and the successfully treated period can be mismatched. During the period of blur removal, myopization can synchronize in the amblyopic eye and increase in the fellow eye.

Previous studies suggest that a higher magnitude of anisometropia is associated with a worse visual function and the severity of amblyopia. Specifically, a large anisometropia (3 D or more) can perturb stereoacuity and binocular fusion (45). Our findings show that the refractive error change in the amblyopic eye is significantly greater than that in the fellow eye, contributing to the reduction of anisometropia. Figure 3 indicates that there is a remaining refractive error of 4.57 ± 1.40 D in the amblyopic eye and anisometropia of 3.64 ± 1.22 D. The finding indicates that spectacle prescriptions are necessary for an extended period.

In conclusion, we demonstrate that the amblyopic eye experiences a significantly greater myopization than the fellow eye in anisometropic amblyopes. The previous amblyopic eye showed a similarly refractive error change between the amblyopic period and the resolving period. The myopization of the fellow eye showed an increase after patching therapy was terminated. We also found a continuous refractive error change before and after 7 years old.

## Data availability statement

The raw data supporting the conclusions of this article will be made available by the authors, without undue reservation.

## Ethics statement

The studies involving human participants were reviewed and approved by the Human Ethics Committees at the Eye Hospital of Wenzhou Medical University. Written informed consent from the participants' legal guardian/next of kin was not required to participate in this study in accordance with the national legislation and the institutional requirements.

## Author contributions

YC, JZh, and HC designed the study. YC and JZu analyzed and interpreted the data and wrote the manuscript. All authors provided a final review and approved the manuscript before submission.

## Funding

This study was supported by the National Natural Science Foundation of China (Grant NSFC 31970975), the Natural Science Foundation for Distinguished Young Scholars of Zhejiang Province, China (LR22H120001), the Project of State Key Laboratory of Ophthalmology, Optometry and Vision Science, Wenzhou Medical University (No. J02-20210203) to JZh, and the Zhejiang Provincial Leading Health Talent Project (HC).

## Conflict of interest

The authors declare that the research was conducted in the absence of any commercial or financial relationships that could be construed as a potential conflict of interest.

## Publisher's note

All claims expressed in this article are solely those of the authors and do not necessarily represent those of their affiliated organizations, or those of the publisher, the editors and the reviewers. Any product that may be evaluated in this article, or claim that may be made by its manufacturer, is not guaranteed or endorsed by the publisher.
